# Effects of mean arterial pressure target on mottling and arterial lactate normalization in patients with septic shock: a post hoc analysis of the SEPSISPAM randomized trial

**DOI:** 10.1186/s13613-022-01053-1

**Published:** 2022-08-19

**Authors:** Nicolas Fage, Julien Demiselle, Valérie Seegers, Hamid Merdji, Fabien Grelon, Bruno Mégarbane, Nadia Anguel, Jean-Paul Mira, Pierre-François Dequin, Soizic Gergaud, Nicolas Weiss, François Legay, Yves Le Tulzo, Marie Conrad, Remi Coudroy, Frédéric Gonzalez, Christophe Guitton, Fabienne Tamion, Jean-Marie Tonnelier, Jean Pierre Bedos, Thierry Van Der Linden, Antoine Vieillard-Baron, Eric Mariotte, Gaël Pradel, Olivier Lesieur, Jean-Damien Ricard, Fabien Hervé, Damien Du Cheyron, Claude Guerin, Alain Mercat, Jean-Louis Teboul, Peter Radermacher, Pierre Asfar

**Affiliations:** 1 Department of Medical Intensive Care, University Hospital of Angers, Angers, France; 2 MITOVASC Laboratory UMR INSERM (French National Institute of Health and Medical Research), 1083 – CNRS 6015, University of Angers, Angers, France; 3 Department of Intensive Care (Service de Médecine Intensive – Réanimation), Nouvel Hôpital Civil, University Hospital of Strasbourg, Strasbourg, France; 4 INSERM (French National Institute of Health and Medical Research), UMR 1260, Regenerative Nanomedicine (RNM), FMTS (Fédération de Médecine Translationnelle de Strasbourg), University of Strasbourg, Strasbourg, France; 5 Service de Biométrie, Institut de Cancérologie de L’Ouest, Centre Paul Papin, Angers, France; 6 Medical and Surgical Intensive Care Unit, Le Mans Hospital, Le Mans, France; 7 Department of Medical and Toxicological Critical Care, Lariboisière Hospital, Paris University, INSERM UMRS-1144, Paris, France; 8 Department of Medical Intensive Care, Bicêtre University Hospital, AP-HP, Paris-Saclay University, Le Kremlin Bicêtre, France; 9 Department of Medical Intensive Care, Cochin University Hospital, Paris, France; 10 Department of Medical Intensive Care, Tours University Hospital, Tours, France; 11 Department of Surgical Intensive Care, University Hospital of Angers, Angers, France; 12 Department of Medical Intensive Care, Georges Pompidou European Hospital, Assistance Publique – Hôpitaux de Paris, University of Paris, Paris, France; 13 Medical and Surgical Intensive Care Unit, Saint Brieuc Hospital, Saint Brieuc, France; 14 Department of Infectious Diseases and Medical Intensive Care, Rennes University Hospital, Rennes, France; 15 Department of Medical Intensive Care, Nancy University Hospital, Nancy, France; 16 Department of Medical Intensive Care, Université de Poitiers, CHU Poitiers, Poitiers, France; 17 Department of Medical and Surgical Intensive Care, Avicenne Teaching Hospital, Bobigny, France; 18 Department of Medical Intensive Care, Nantes University Hospital, Nantes, France; 19 Department of Medical Intensive Care, Rouen University Hospital, Rouen, France; 20 Department of Medical Intensive Care, Brest University Hospital, Brest, France; 21 Intensive Care Unit, Versailles Hospital, Le Chesnay, France; 22 Department of Intensive Care, Saint Philibert Hospital, Catholic University of Lille, Lille, France; 23 Department of Medical Intensive Care, University Hospital of Ambroise Paré, Boulogne Billancourt, France; 24 Inserm U1018, Center for Research in Epidemiology and Population Health (CESP), Faculty of Paris Saclay, Villejuif, France; 25 Department of Intensive Care, Saint Louis Hospital, Paris, France; 26 Department of Intensive Care, Avignon Hospital, Avignon, France; 27 Department of Medical and Surgical Intensive Care, La Rochelle Saint Louis Hospital, La Rochelle, France; 28 Université de Paris, AP-HP, Hôpital Louis Mourier, DMU ESPRIT, Médecine Intensive Réanimation, Colombes, France; 29 Department of Medical and Surgical Intensive Care, Quimper Hospital, Quimper, France; 30 Department of Medical Intensive Care, Caen University Hospital, Caen, France; 31 Department of Medical Intensive Care, Edouard Herriot Hospital, Lyon, France; 32 Institut für Anästhesiologische Pathophysiologie und Verfahrensentwicklung, Universitätsklinikum, Helmholtzstrasse 8-1, Ulm, Germany

**Keywords:** Arterial lactate, Lactate clearance, Mean arterial pressure, Microcirculation, Mottling, Septic shock

## Abstract

**Background:**

In patients with septic shock, the impact of the mean arterial pressure (MAP) target on the course of mottling remains uncertain. In this post hoc analysis of the SEPSISPAM trial, we investigated whether a low-MAP (65 to 70 mmHg) or a high-MAP target (80 to 85 mmHg) would affect the course of mottling and arterial lactate in patients with septic shock.

**Methods:**

The presence of mottling was assessed every 2 h from 2 h after inclusion to catecholamine weaning. We compared mottling and lactate time course between the two MAP target groups. We evaluated the patient’s outcome according to the presence or absence of mottling.

**Results:**

We included 747 patients, 374 were assigned to the low-MAP group and 373 to the high-MAP group. There was no difference in mottling and lactate evolution during the first 24 h between the two MAP groups. After adjustment for MAP and confounding factors, the presence of mottling ≥ 6 h during the first 24 h was associated with a significantly higher risk of death at day 28 and 90. Patients without mottling or with mottling < 6 h and lactate ≥ 2 mmol/L have a higher probability of survival than those with mottling ≥ 6 h and lactate < 2 mmol/L.

**Conclusion:**

Compared with low MAP target, higher MAP target did not alter mottling and lactate course. Mottling lasting for more than 6 h was associated with higher mortality. Compared to arterial lactate, mottling duration appears to be a better marker of mortality.

**Supplementary Information:**

The online version contains supplementary material available at 10.1186/s13613-022-01053-1.

## Background

Septic shock is characterized by macro- and micro-circulatory impairments related to an infection. This condition is associated with a high mortality rate, above 40% according to the SEPSIS-3 criteria [1].

The microcirculatory impairment is a key pathophysiological point and results from several mechanisms (endothelial dysfunction, impaired inter-cell communication, altered glycocalyx, adhesion and rolling of white blood cells as well as platelets, and altered red blood cell deformability) [2]. The persistence of microcirculation dysfunctions during septic shock, assessed by sublingual microvascular damage, is associated with a higher mortality [3].

Both mottling and arterial lactate have been validated as prognostic markers of mortality [1, 4, 5]. Mottling can be easily assessed at the bedside [5, 6]. Its severity as quantified using the mottling score (evaluated 6 h after admission to the intensive care unit), is strongly related to 14-day and 28-day mortality [4, 7, 8]. Moreover, an improvement in mottling score and a shorter duration of mottling are associated with a higher survival at day 28 [4, 8, 9]. Arterial lactate is considered as the mirror of tissue hypoxia and has been validated as a prognostic marker of mortality [10–12].

During septic shock, a dissociation between macrocirculatory hemodynamics and microcirculatory alterations has been reported [13]. Indeed, some patients experience a multiple organ failure with a persistence of microcirculation dysfunctions despite an improvement in systemic hemodynamics [9]. To date, only a few studies involving a small number of patients have assessed the impact of mean arterial pressure (MAP) target on microcirculation alterations, with conflicting results and heterogeneities in the timing of interventions [14–16].

In the SEPSISPAM trial [17], a high-MAP target (80 to 85 mmHg) was compared to a low-MAP target (65 to 70 mmHg), determined at the early phase of septic shock (i.e. within the 6 h following vasopressor introduction). In this post hoc analysis, we investigated whether, in patients with septic shock, different MAP targets may impact the time course of skin mottling, considered as an acceptable surrogate of microcirculation status and/or of arterial lactate normalization, considered as a marker of tissue hypoxia.

## Materials and methods

### Patient selection

In the randomized SEPSISPAM trial [17], patients were enrolled from 29 centers in France. Randomization was performed with the use of a computer-generated assignment sequence in a centralized, blinded fashion and was stratified according to whether patients had chronic arterial hypertension (i.e., had been receiving antihypertensive treatment or had a history of chronic arterial hypertension). Patients older than 18 years of age were enrolled if they had septic shock (according to SEPSIS-2 definition), if they required vasopressors at a minimum infusion rate of 0.1 µg per kilogram per minute, and if they were evaluated within 6 h after the initiation of vasopressor. After enrollment, patients were assigned to vasopressor treatment that was adjusted to maintain a MAP of 80 to 85 mmHg (high-MAP target group) or 65 to 70 mmHg (low-MAP target group). The MAP target was maintained for a maximum of 5 days or until the patient was weaned from vasopressor support.

For this post hoc analysis of the SEPSISPAM trial [17], we included patients with at least one available data regarding mottling. Data that concerned mottling were considered until the discontinuation of catecholamine or for a maximum of 5 days under vasopressors.

### Data collection

In the SEPSISPAM trial, the presence or absence of mottling was assessed by nurses every two hours, from 2 h after the inclusion to the 5 first days or to the discontinuation of catecholamine. In patients weaned from catecholamine for the 5 first days, the presence or absence of mottling was assessed up to 12 h after norepinephrine weaning. Skin mottling was defined as a red-violaceous discoloration of skin on the knee or above it. No information was collected regarding mottling intensity (mottling score). For each patient, we calculated the duration of mottling over the first 24 h after inclusion.

Arterial lactate levels were measured at inclusion, 6 and 12 h after inclusion and once daily thereafter. An arterial lactate level lower than 2 mmol/L was considered as normal.

### Outcomes

The primary outcome was the mottling duration for the first 24 h after inclusion. Secondary outcomes were the time to normalization of arterial lactate and mortality at day 28 and day 90.

### Ethical concerns

The SEPSISPAM trial (NCT01149278) was approved for all participating centers by the ethic committee at the Angers University Hospital. Written inform consent was obtained from all patients, their next of kin or another surrogate decision-maker, as appropriate. In accordance with ethics policy, if patients were unable to provide informed consent and the next of kin or a designated person was not available, the emergency inclusion procedure was applied and post hoc consent was obtained from patients who survived.

### Statistical analysis

Quantitative variables, presented as median [interquartile range] were compared with Mann–Whitney test. Qualitative variables, presented as the absolute value [percentage] were compared with Fisher’s exact test. Survival curves were computed by the Kaplan–Meier method and compared by the log-rank test. First, only available data were analyzed, then, we re-analyzed the dataset of mottling, dealing with missing data, assuming that when mottling state was similar around missing data, the mottling state for the missing data was the same (last observation carried forward: LOCF). For instance, if the patient has mottling at H2 and H6, and the data were missing at H4, then the patient was considered with mottling at H4.

A mixed model was used to explore the evolution of MAP during the 5-day study period according to the MAP target.

A mixed effect logistic regression was used to explore the relation between time course of mottling, MAP target and history of chronic arterial hypertension.

Cox regression models were computed to explore the relation between mottling and mortality at day 90. In the first step, univariate analyses were conducted separately for each inclusion characteristic. In the second step, multivariate Cox regression models were built using variables with *p*-value < 0.05 in the univariate analyses: age, SAPS II, history of ischemic heart disease and of chronic arterial hypertension, source of infection, MAP, lactate, fluid intake, vasopressor dose and need for mechanical ventilation at inclusion. We add the randomization group in the model. We did not include the pH, SOFA, acute kidney injury or PaO_2_/FiO_2_ ratio because of their redundancy with other variables. Results were presented as hazard ratio (HR) with 95% confidence intervals (95% CI).

Statistical analysis was performed using Prism GraphPad Software v3.9.1 (San Diego, California) and R (R Foundation for Statistical Computing, Vienna, Austria. URL https://www.R-project.org/) v3.6.2. All tests were two-sided, and p-values below 0.05 were considered as statistically significant.

## Results

### Study population

Thirty-three patients were excluded because of missing data regarding mottling. Seven hundred and forty-seven patients were thus included in the analysis. Among them, 374 and 373 patients were assigned to the low-MAP target group and the high-MAP target group, respectively (Additional file 2: Fig. S1). Baseline characteristics and mortality at day 90 were similar in the low and high-MAP target groups (Additional file 1: Table S1). MAP values measured during the first 5 days were significantly lower in the low-MAP target group than in the high-target MAP group (*p* < 0.001, Additional file 2: Fig. S2).

Patients in the high-MAP target group received higher doses of norepinephrine during the first 5 days than patients in the low-MAP target group. Fluid intake was similar in both groups (Additional file 1: Table S2).

Among the 747 analyzed patients, 296 (40%) experienced mottling for at least 2 h during the first 24 h after inclusion. The median duration of mottling during the first 24 h after inclusion was 14 [1, 6–22] h. Among patients with mottling, 235 (79.4%) had mottling that lasted more than 6 h.

The whole mottling monitoring (i.e. every 2 h in patients with catecholamines infusion) represented 21,544 timepoints. Data were missing at 610 timepoints (2.8% of the entire dataset), and, after extrapolation with the LOCF procedure, only 40 timepoints data remained missing (0.2% of the dataset).

As presented in Table 1, disease severity was higher in patients with mottling during the first 24 h than in those without mottling (higher Simplified Acute Physiology Score II (SAPS II) [63 (50–77) vs. 53 (43–63), *p* < 0.0001] and higher Sequential Organ Failure Assessment (SOFA) scores [11 (9–13) vs. 10 (8–12), *p* < 0.0001]). Patients with mottling had more comorbidities and worse hemodynamic parameters at baseline (MAP, heart rate, arterial lactate). Patients with mottling required higher levels of vasopressor and had higher mortality at day 28 and day 90 (Additional file 1: Table S3).

**Table 1:** Baseline characteristics of study patients according to the presence or absence of mottling at baseline or during the first 24 h

Characteristic	No mottling *n* = 451	Mottling *n* = 296	*p*-value
Age—years	65 [54–75]	69 [58–78]	**0**.**002**
Male sex	302 [67]	201 [67]	0.81
SAPS II	53 [43–63]	63 [50–77]	**< 0**.**0001**
SOFA	10 [8–12]	11 [9–13]	**< 0**.**0001**
Pre-existing condition—no (%)			
Ischemic heart disease	35 [8]	43 [14]	**0**.**003**
Chronic heart failure	55 [12]	53 [18]	**0**.**03**
COPD	63 [14]	39 [13]	0.83
Chronic kidney disease	49 [11]	42 [14]	0.21
Cirrhosis	29 [6]	23 [8]	0.56
Chronic arterial hypertension	208 [46]	166 [57]	**0**.**008**
Cancer or autoimmune disease	167 [37]	97 [33]	0.24
Source of infection—no (%)			
Lung	252 [56]	136 [46]	**0**.**009**
Abdomen	60 [13]	66 [22]	**0**.**001**
Urinary tract	59 [13]	26 [9]	0.08
Others	80 [18]	68 [23]	0.09
Community-acquired infection—no (%)	296 [66]	199 [67]	0.69
Hemodynamic and biochemical variable			
Mean arterial pressure -mmHg	74 [65–83]	71 [61–81]	**0**.**01**
Heart rate—beats/min	100 [81–117]	107 [88–126]	**< 0**.**0001**
Arterial pH	7.34 [7.28–7.39]	7 .25 [7.15–7.35]	**< 0**.**0001**
Serum lactate level—mmol/L	2 [1.3–3.1]	3.2 [1.8–5.4]	**< 0**.**0001**
Fluid therapy before inclusion—mL	2500 [2000–3250]	3000 [2500–4000]	**< 0**.**0001**
Vasoactive drug infusions at randomization—no (%)			
Norepinephrine	434 [96]	274 [93]	**0**.**04**
Dobutamine	21 [5]	20 [7]	0.25
Epinephrine	18 [4]	36 [12]	**0**.**0004**
Median vasopressor dose at randomization—µg/kg/min			
Norepinephrine	0.32 [0.2–0.52]	0.44 [0.22–0.84]	**0**.**0004**
Epinephrine	0.34 [0.16–0.57]	0.21 [0.14–0.5]	0.35
Mechanical ventilation—no (%)	317 [70]	253 [85]	**< 0**.**0001**
PaO_2_/FiO_2_ ratio -mmHg	178 [122–267]	158 [90–245]	**0**.**0098**
Acute kidney injury—no (%)	175 [39]	169 [57]	**< 0**.**0001**

Patients in the “no mottling” group had no mottling during the first 24 h of septic shock. Patients in the mottling group had mottling for at least 2 h during the first 24 h of septic shock

COPD: chronic obstructive pulmonary disease

Values are represented as median [interquartile range]. The target mean arterial pressure was 80 to 85 mmHg in the high-target group and 65 to 70 mmHg in the low-target group

The Simplified Acute Physiology Score (SAPS) II is based on 17 variables and scores range from 0 to 163, with a higher score indicating a more severe disease

The score on the Sequential Organ Failure Assessment (SOFA) includes sub-scores ranging from 0 to 4 for each of five components (circulation, lungs, liver, kidneys and coagulation). Aggregated scores range from 0 to 20, with higher scores indicating more severe organ failure

Others sources of infection included blood, soft tissue, skin, central venous system, bones and joints, cardiac system, reproductive organs and unknown sources

Acute kidney injury was defined as a renal SOFA score of 2 or more (plasma creatinine level > 1.9 mg/dL (168 µmol/L) or urinary output, < 500 mL per day)

Patients with mottling had higher arterial lactate levels at inclusion than patients without mottling [3.2 (1.8–5.4) vs. 2.0 (1.3–3.1), *p* < 0.0001], without difference between MAP-target groups (Additional file 2: Fig. S3*).* The duration of mottling during the first 24 h was higher in patients with an arterial lactate level ≥ 2 mmol/L at inclusion than in patients with an arterial lactate level < 2 mmol/L [[0.0 (0.0–14.0) h] vs 0.0 (0.0–2.0) h, *p* < 0.0001]. The time required to normalize arterial lactate was shorter in patients without mottling than in those with mottling (*p* < 0.0001, Additional file 2: Fig. S4).

### Impact of MAP target on mottling and arterial lactate

**Fig. 1:**
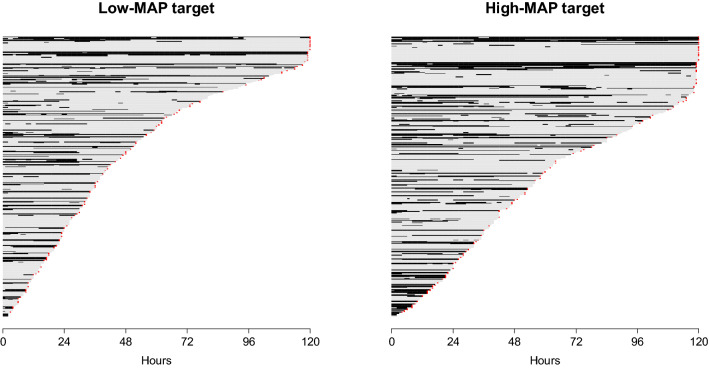
Course of mottling in patients with septic shock, according to the mean arterial pressure (MAP) target. Low-MAP target group: 65–70 mmHg, high-MAP target group: 80–85 mmHg. Each horizontal line represents a patient follow-up. The length of the line represents the length of observation time of the patient. Black line corresponds to a period of time with mottling; and grey line corresponds to a period of time without mottling for the patient. There was no difference in mottling time course according to the MAP target. A red asterisk at the end of a line represents the death of the patient

**Fig. 2:**
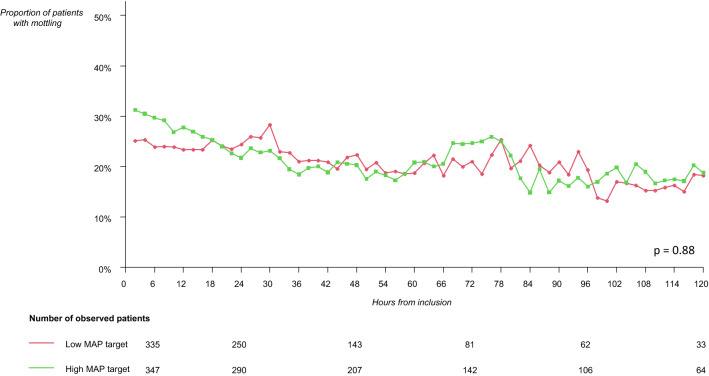
Evolution of the proportion of patients with mottling according to the mean arterial pressure (MAP) target during septic shock. Low-MAP target group: 65–70 mmHg, high-MAP target group: 80–85 mmHg. This figure represents the proportion of patients with mottling (number of patients with mottling out of the number of patients observed) at each time. There was no difference in the evolution of proportion of patients with mottling during septic shock according to the MAP target (Wilcoxon test: *p* = 0.88)

There was no difference in mottling duration during the first 24 h between low and high-MAP target groups (Fig. 1), nor in the proportion of patients with mottling while under vasopressors according to MAP target (Fig. 2, Additional file 1: Table S4). Among the 747 patients, 519 were weaned from catecholamines during the first 5 days. There was no difference in mottling duration after weaning of norepinephrine between low [0 (0–0) h) and High-MAP target group [0 (0–0) h, *p* value = 0.68] (Additional file 1: Table S4) and mottling during period of weaning was observed only in 21 patients in the low-MAP target group and in 16 patients in the high-MAP target group (*p* value = 0.74). In both groups of patients with catecholamines and weaned from catecholamines, there was no difference in the proportion of patients with or without mottling at H24, H48 and H72 of the inclusion according to the MAP target (Additional file 1: Table S4).

**Fig. 3:**
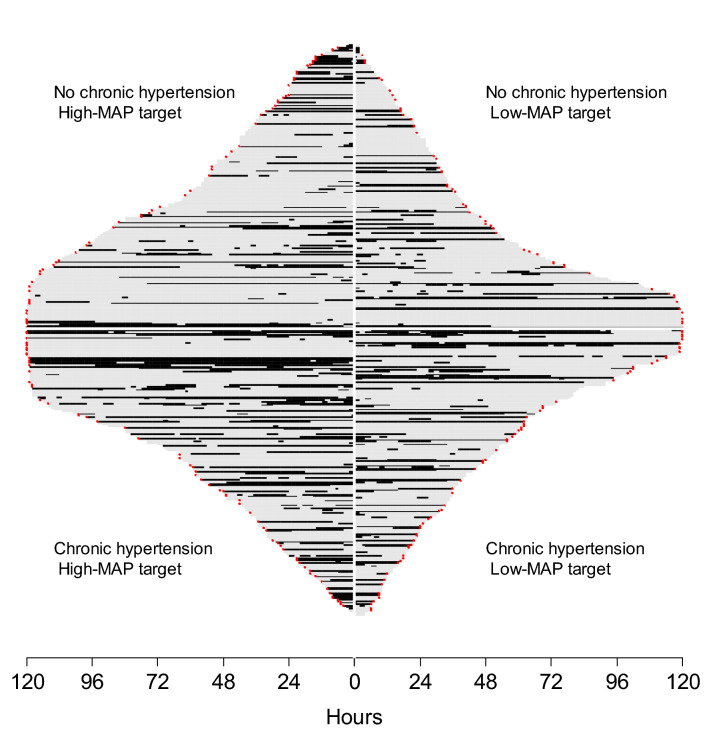
Course of mottling in patients with septic shock, according to the mean arterial pressure (MAP) target and chronical arterial hypertension. Low-MAP target group: 65–70 mmHg, High-MAP target group: 80–85 mmHg. Each horizontal line represents a patient follow-up. Solid line corresponds to a period of time with mottling; and hatched line corresponds to a period of time without mottling for the patient. A red asterisk at the end of a line represents the death of the patient. There was no difference in mottling time course according to the MAP target and chronic arterial hypertension

Furthermore, no difference was observed in the duration of mottling according to the history of chronic arterial hypertension and MAP target during the first 24 h (Fig. 3). Indeed there was no significant association between the probability of observing mottling according to the MAP target (*p* = 0.369) and to the history of chronic arterial hypertension (*p* = 0.144). Our results were similar when considering only patients who met the criteria of the SEPSIS-3 definition of septic shock (Additional file 2: Fig. S5) and when considering mottling with the LOCF procedure (Additional file 2: Figs. S6 and S7).

In addition, in patients with elevated arterial lactate at inclusion (according to the SEPSIS-3 definition [1]), there was no difference in the time course changes of arterial lactate concentrations between the two MAP-target groups (Additional file 2: Fig. S8).

### Association between mottling and mortality

**Fig. 4:**
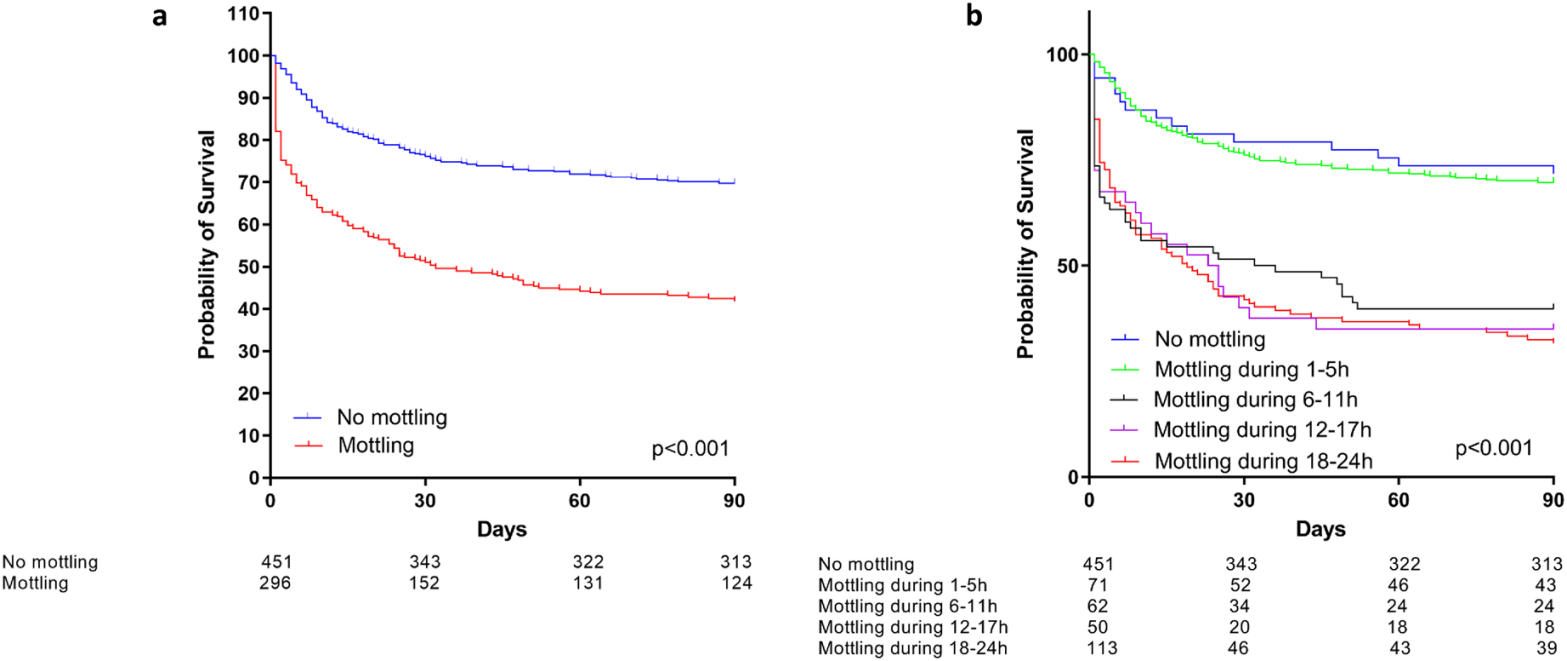
Survival according to the presence (mottling) or absence of mottling (no mottling) during the first 24 h after inclusion (**a**) and according to the duration of mottling (**b**). **a** *p*-value refers to the difference between patients with mottling and those without. Log rank test: *p* < 0.0001. **b** *p*-value refers to the difference between each subgroup of mottling duration

The presence of mottling within the first 24 h after inclusion was associated with a significant increase in the risk of death at day 90 (log-rank test *p* < 0.001) (Fig. 4a). Similar results were found when considering only patients who met the criteria of the SEPSIS-3 definition of septic shock [1].

When compared to patients with mottling lasting for more than 6 h, patients without mottling or with mottling lasting for less than 6 h had a lower risk of death at day 90 (Fig. 4b and Table 2). In Table 2, the adjustment of the multivariate analysis was performed on the MAP target and on differences between patients at baseline (age, SAPS II, history of chronic arterial hypertension or of myocardial infarction, source of infection and MAP, fluid intake, vasopressor dose and need for mechanical ventilation at inclusion).

**Table 2:** Univariate and multivariate analysis evaluating the impact of the course of mottling on day 90 mortality in septic shock

Duration of mottling within the first 24 h	Univariate analysis *n* = 747		Multivariate analysis^†^ *n* = 656	
	HR	*p*-value	HR	*p*-value
No mottling (reference)	1	–	1	–
1–5 h	1.24 [0.79–1.92]	0.35	1.03 [0.62–1.72]	0.91
6–24 h	3.32 [2.64–4.18]	< 0.001	2.14 [1.63–2.81]	< 0.001
1–5 h (reference)	1	–	1	–
6–24 h	2.69 [1.73–4.17]	< 0.001	2.08 [1.25–3.46]	0.005

^†^Multivariate analysis based on the randomization group (low-MAP or high-MAP target), lactate at inclusion, age, SAPS II, history of chronic arterial hypertension, history of ischemic heart disease, and MAP, fluid intake, vasopressor dose and need for mechanical ventilation at inclusion. We did not include the pH, SOFA, acute kidney injury or PaO_2_/FiO_2_ ratio for competing with other variables. Ninety-one patients were excluded because of missing data on at least 1 adjustment variable

**Fig. 5:**
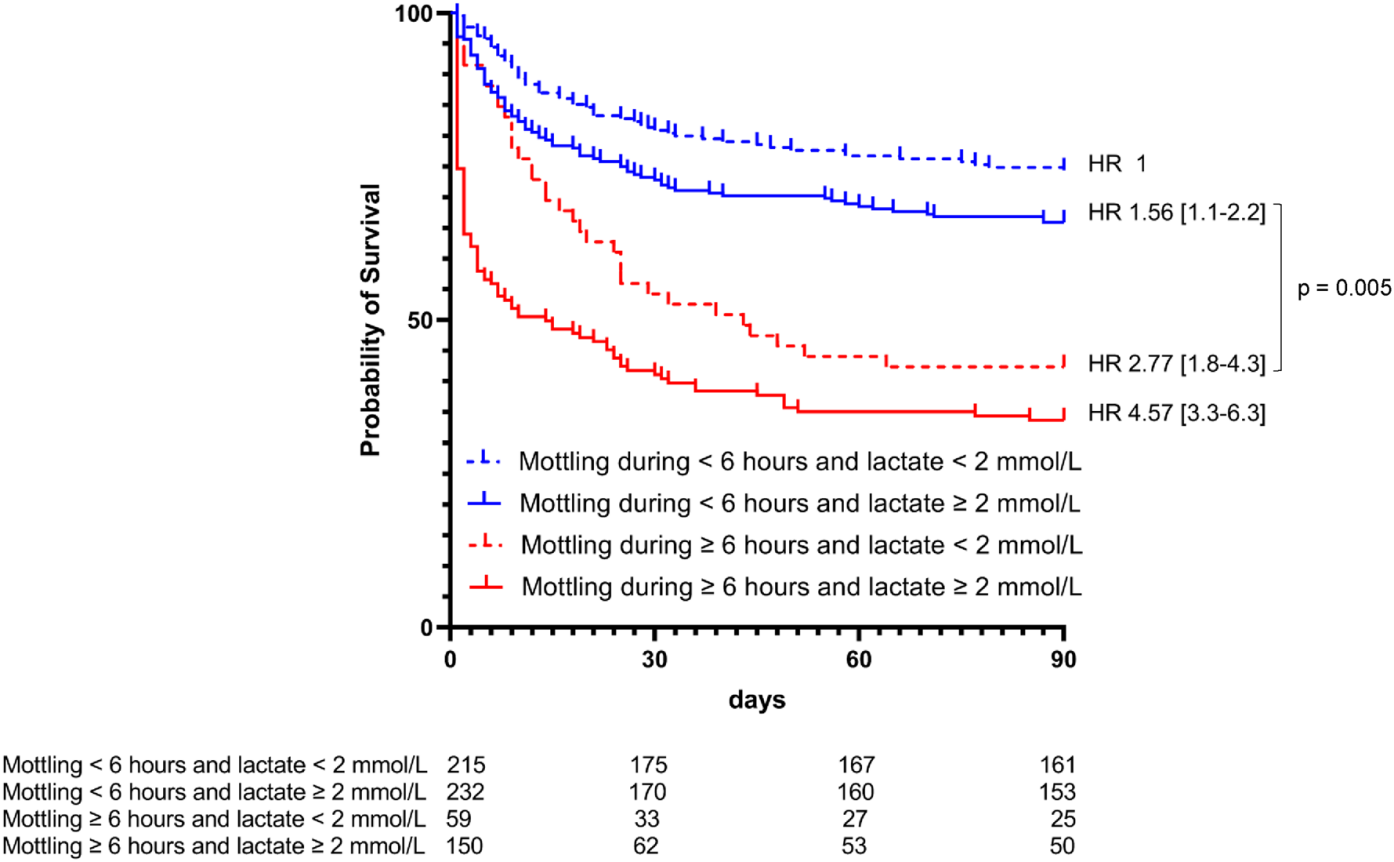
Survival according to mottling duration during the first 24 h and arterial lactate at inclusion. Kaplan–Meier curves represent the survival according to the duration of mottling and the arterial lactate level. Hazard ratios (HR) were calculated using a Cox model. We defined the group of patients with mottling duration < 6 h during the first 24 h of septic shock and lactate < 2 mmol/L at inclusion as the reference. HR were adjusted on randomization group, age, SAPS II, past medical history (chronic arterial hypertension, myocardial infarction,) and MAP, lactate, fluid intake, vasopressor dose at inclusion and need for mechanical ventilation at inclusion. Patients without mottling or mottling lasting for less than 6 h and with arterial lactate ≥ 2 mmol/L had a higher probability of survival than patients with mottling lasting for ≥ 6 h and arterial lactate < 2 mmol/L (*p* = 0.005)

Figure 5 illustrates the respective weight of mottling duration and arterial lactate level on mortality: patients without mottling or mottling lasting for less than 6 h and with arterial lactate ≥ 2 mmol/L have a higher probability of survival as compared with patients with mottling lasting for more than 6 h and arterial lactate < 2 mmol/L (*p* = 0.005). Indeed, 153 of the 232 patients without mottling or mottling lasting for less than 6 h and with arterial lactate ≥ 2 mmol/L were alive at day 90 as compared to 25 of the 59 patients with mottling lasting during for more than 6 h and arterial lactate < 2 mmol/L.

## Discussion

In this post hoc analysis of the SEPSISPAM trial, the course of mottling was not found to be associated with the MAP target level (low-MAP versus high-MAP target) or with the time course of arterial lactate levels. In addition, we confirmed the strong relationship between mottling duration ≥ 6 h and mortality. Moreover, the multivariate analysis showed that the presence of mottling during the first 24 h was more strongly associated with mortality than the arterial lactate level.

In patients with septic shock, increasing MAP target in patients with chronic arterial hypertension may reduce the need for renal replacement therapy [17]. However, the increase in doses of norepinephrine to achieve a higher MAP target may also increase the risk of tissue ischemia [18]. Our results show that increasing MAP in patients with septic shock is safe regarding microcirculation.

Microcirculation impairment and its dissociation from macrocirculatory hemodynamics is one of the cornerstones of the pathophysiology of septic shock [3]. Results of previous studies regarding the effect of MAP level on microcirculation are conflicting. Small sample size studies reported an improvement of the sublingual or cutaneous microcirculation with an increase in MAP target [14, 19, 20], while others did not identify any difference between two levels of MAP [16]. Dubin et al. reported a large variability in microvascular responses with different levels of MAP [16]. Our study provides new information on the dissociation between micro- and macro-circulation during septic shock [3], as we found no difference in mottling course and arterial lactate normalization between the two MAP target groups in a large number of patients, with a homogeneous definition of septic shock (according to the SEPSIS-2 definition) and a strong external validity (mortality rate) [21].

Of note, the design of the SEPSISPAM trial and our post hoc analysis did not allow to explain the pathophysiological impairment of microcirculatory blood flow. Whether mottling is more than a microcirculation surrogate and may convey a broader array of underlying mechanisms (adrenergia, endothelial dysfunction, microthrombosis, microcirculatory low flow, etc.) remains unanswered.

Beyond its interest as an acceptable marker of microcirculation impairment, mottling has already been reported to be a marker of mortality [4, 7]. Ait-oufella et al*.* described the association between mottling and its intensity (through a semi-quantitative approach, namely the mottling score) and worse outcomes in 60 patients [4]. In addition, mottling duration (more than 6 h despite adequate resuscitation) was reported to be predictive of death at day 14 [4, 9] and day 28 [7, 8] in heterogeneous populations. Similar results were found in sepsis [9, 22].

Coudroy et al. [8] also reported a higher mortality of ICU patients with mottling lasting more than 6 h. In this study, only 65 out of 791 patients had, however, been referred to ICU for septic shock [8]. Our results confirm the poor outcomes of patients with mottling in a well-defined population of septic shock according to SEPSIS-2 [23] and SEPSIS-3 definitions [1]. Furthermore, we confirm on a larger scale that patients with mottling lasting less than 6 h have the same outcomes than those without mottling.

Our study shows a higher mortality in patients with mottling lasting for more than 6 h without hyperlactatemia as compared to patients without mottling and with arterial lactate ≥ 2 mmol/L at inclusion. These results are consistent with the analysis of Ait-oufella et al. [4] where the mottling score was a stronger predictor of mortality than arterial lactate. These results were confirmed by Dumas et al. [9]. The better ability of mottling to predict death could be explained by the fact that arterial lactate is the result of a balance between peripheral tissue production and several pathogenic mechanisms (stress-related adrenergic-induced glycolysis, impaired hepatic lactate clearance) [24, 25], whereas skin mottling is probably a more direct assessment of microcirculatory impairment. However, skin mottling is not the only marker of microcirculation impairment. Brunauer et al. reported that mottling intensity was less correlated to visceral organ pulsatility indices than capillary refill time [26].

Our study has several limitations. First of all, the post hoc design is well known for its biases. Second, the data regarding mottling collected in the SEPSISPAM trial consisted only of the presence or absence of mottling, without information on the intensity and extent of mottling. Such information, namely the mottling score [4], could have been of interest in our study, to better assess whether MAP target may have an impact on mottling score intensity. However, discriminating patients with mottling from those without decreases the risk of interobserver variability and increases the robustness of mottling assessment. Third, as in the SEPSISPAM trial, achieved MAP was higher than targeted MAP, although there was still a significant difference in MAP between the low-target and high-target MAP groups. Finally, we acknowledge that a delayed impact of higher vasopressor dose could have been missed due to the end of the monitoring of mottling. There was no difference between duration of mottling after weaning of norepinephrine in both groups as mottling was assessed up to 12 h after norepinephrine weaning. As the half-life of norepinephrine is short, a persistent late effect seems unlikely.

## Conclusion

This study shows that in patients with septic shock, a MAP target between 80 and 85 mmHg, achieved through increased vasopressor doses, did not alter the course of mottling nor lactate normalization. This study confirms that the presence of mottling lasting for ≥ 6 h was associated with a higher mortality at day 28 and 90 in these patients. In addition, compared to the arterial lactate level at inclusion, mottling duration appears to be a stronger marker of mortality risk. Our results suggest that a deeper pathophysiological understanding of mottling is still pending.

## Supplementary Information

**Additional file 1.** Additional Tables.

**Additional file 2.** Additional Figures.

## Data Availability

The datasets analyzed during the current study are available from the corresponding author on reasonable request.

## Abbreviations

LOCF: Last observation carried forward
MAP: Mean arterial pressure
HR: Hazard ratio
CI: Confidence intervals
SAPS II: Simplified Acute Physiology Score II
SOFA: Sequential Organ Failure Assessment
