# Supplementary material for: Effects of mean arterial pressure target on mottling and arterial lactate normalization in patients with septic shock: a post hoc analysis of the SEPSISPAM randomized trial
Source: Ann Intensive Care. 2022 Aug 19;12:78. doi: 10.1186/s13613-022-01053-1 (PMC9391564; doi:10.1186/s13613-022-01053-1)
Supplement: Supplementary file 1 — Additional file 1. Additional Tables. [file 13613_2022_1053_MOESM1_ESM.docx]

**SUPPLEMENTARY MATERIAL**

**Tables of contents**

[**SUPPLEMENTAL FIGURES** 2](#_Toc109727859)

[**Figure S1 Flow chart of the study** 2](#_Toc109727860)

[**Figure S2 Mean Arterial Pressure during the 5-day study period according to level of MAP target** 2](#_Toc109727861)

[**Figure S3 Relation between presence or absence of mottling in the first 24 hours, mean arterial pressure (MAP) target and arterial lactate at inclusion** 2](#_Toc109727862)

[**Figure S4 Arterial lactate normalization according to presence or absence of mottling in the first 24 hours** 2](#_Toc109727863)

[**Figure S5 Course of mottling in patients with septic shock and lactate ≥ 2 mmol/L (SEPSIS 3 definition), according to the mean arterial pressure (MAP) target and chronical hypertension** 2](#_Toc109727864)

[**Figure S6 Course of mottling in patient with septic shock, according to the mean arterial pressure (MAP) target, with interpolation of missing data.** 3](#_Toc109727865)

[**Figure S7 Course of mottling in patient with septic shock, according to the mean arterial pressure (MAP) target and chronical hypertension, with interpolation of missing data.** 3](#_Toc109727866)

[**Figure S8 Arterial lactate normalization according to the MAP target** 4](#_Toc109727867)

[**SUPPLEMENTALS TABLES** 5](#_Toc109727868)

[**Table S1 Baseline characteristics of study patients according to MAP target.** 6](#_Toc109727869)

[**Table S2 Evolution of norepinephrine doses and fluid intake according to the MAP target** 7](#_Toc109727870)

[**Table S3 Outcomes according to the presence or absence of mottling at baseline or during the first 24 hours** 8](#_Toc109727871)

[**Table S4 Proportion of patients with mottling at H24, H48 and H72 of the inclusion.** 8](#_Toc109727872)

# **SUPPLEMENTAL FIGURES**

## **Figure S1 Flow chart of the study**

*SEPSISPAM trial refer to* (18)*. MAP: Mean arterial pressure.*

## **Figure S2 Mean Arterial Pressure during the 5-day study period according to level of MAP target**

*Redline : High-MAP target, black line : Low-MAP target. Values are presented as mean with Confidence Intervals (I bars).*

*p value < 0.001 (*calculated from a mixed model).

## **Figure S3 Relation between presence or absence of mottling in the first 24 hours, mean arterial pressure (MAP) target and arterial lactate at inclusion**

*There is no difference between High-MAP target and Low-MAP target (ns, not significant: p value > 0.05).*

*MAP: Mean arterial pressure.*

## **Figure S4 Arterial lactate normalization according to presence or absence of mottling in the first 24 hours**

*Log rang test p <0*.*001.*

## **Figure S5 Course of mottling in patients with septic shock and lactate ≥ 2 mmol/L (SEPSIS 3 definition), according to the mean arterial pressure (MAP) target and chronical hypertension**

*Low-MAP target group: 65-70 mmHg, High-MAP target group: 80-85 mmHg.*

*Each horizontal line represents a patient follow-up. The length of the line represents the length of observation time of the patient. Black line corresponds to a period of time with mottling; and grey line corresponds to a period of time without mottling for the patient.*

*A red asterisk at the end of a line represents the death of the patient.*

*There was no difference in mottling time course according to the MAP target and chronical hypertension in the subgroup of SEPSIS-3 definition.*

## **Figure S6 Course of mottling in patient with septic shock, according to the mean arterial pressure (MAP) target, with interpolation of missing data.**

*Low-MAP target group: 65-70 mmHg, High-MAP target group: 80-85 mmHg.*

*Each horizontal line represents a patient follow-up. The length of the line represents the length of observation time of the patient. Black line corresponds to a period of time with mottling; grey line corresponds to a period of time without mottling and white line corresponds to a period with interpolation of the data.*

*There was no difference in mottling time course according to the MAP target and chronical hypertension.*

*A red asterisk at the end of a line represents the death of the patient.*

## **Figure S7 Course of mottling in patient with septic shock, according to the mean arterial pressure (MAP) target and chronical hypertension, with interpolation of missing data.**

*Low-MAP target group: 65-70 mmHg, High-MAP target group: 80-85 mmHg.*

*Each horizontal line represents a patient follow-up. The length of the line represents the length of observation time of the patient. Black line corresponds to a period of time with mottling; grey line corresponds to a period of time without mottling and white line corresponds to a period with interpolation of the data.*

*There was no difference in mottling time course according to the MAP target.*

*A red asterisk at the end of a line represents the death of the patient.*

## **Figure S8 Arterial lactate normalization according to the MAP target**

*Log rang test are not significant (p = 0*.*87).*

*Low-MAP target group: 65-70 mmHg, High-MAP target group: 80-85 mmHg.*

# **SUPPLEMENTALS TABLES**

| **Characteristic** | **Low-MAP target**  n = 374 | **High-MAP target**  n = 373 | **p-value** |
| --- | --- | --- | --- |
| Age – years | 68 [55-77] | 65 [56-75] | 0.5 |
| Male sex | 245 [65.5] | 258 [69.2] | 0.31 |
| SAPS II | 57 [46-68] | 55 [45-69] | 0.26 |
| SOFA | 11 [9-13] | 10 [8-13] | 0.46 |
| Pre-existing condition – no (%) |  |  |  |
| Ischemic heart disease | 39 [10.4] | 39 [10.5] | 0.99 |
| Chronic heart failure | 52 [13.9] | 56 [15] | 0.68 |
| COPD | 46 [12.3] | 56 [15] | 0.29 |
| Chronic kidney disease | 48 [12.8] | 43 [11.5] | 0.65 |
| Cirrhosis | 25 [6.7] | 27 [7.2] | 0.78 |
| Chronic arterial hypertension | 188 [50.3] | 185 [49.6] | 0.94 |
| Cancer or autoimmune disease | 130 [34.8] | 134 [35.9] | 0.76 |
| Source of infection – no (%) |  |  |  |
| Lung | 192 [51.3] | 196 [52.5] | 0.77 |
| Abdomen | 64 [17.1] | 62 [16.6] | 0.86 |
| Urinary tract | 43 [11.5] | 42 [11.3] | 1 |
| Others | 75 [20.1] | 73 [19.6] | 0.93 |
| Community-acquired infection –no (%) | 244 [65.2] | 251 [67.3] | 0.59 |
| Hemodynamic and biochemical variable |  |  |  |
| Mean arterial pressure -mmHg | 72 [63.5-81.5] | 74 [65-83] | 0.18 |
| Heart rate – beats/min | 102 [85-118] | 101 [83-121] | 0.89 |
| Arterial pH | 7**^.^**32 [7.22-7.39] | 7**^.^**32 [7.22-7.38] | 0.94 |
| Serum lactate level –mmol/L | 2 **^.^**4 [1.6-3.87] | 2**^.^**25 [1.4-3.9] | 0.09 |
| Fluid therapy before inclusion - L | 2 **^.^**75 [2-3.5] | 2**^.^**.75 [2-3.5] | 0.71 |
| Vasoactive drug infusions at randomizations – no (%) |  |  |  |
| Norepinephrine | 352 [94.1] | 356 [95.4] | 0.51 |
| Dobutamine | 23 [6] | 18 [4.8] | 0.52 |
| Epinephrine | 32 [8.6] | 22 [5.9] | 0.20 |
| Median vasopressor dose at randomization - µg/kg/min |  |  |  |
| Norepinephrine | 0**^.^**35 [0.2-0.63] | 0**^.^**39 [0.2-0.62] | 0.40 |
| Epinephrine | 0**^.^**23 [0.17-0.52] | 0**^.^**27 [0.15-0.61] | 0.98 |
| Mechanical ventilation - no (%) | 274 [73.3] | 296 [79.4] | 0.06 |
| PaO_2_/FiO_2_ ratio -mmHg | 166 [104-266] | 171 [115-251] | 0.8 |
| Acute kidney injury – no (%) | 182 [48.8] | 162 [43.3] | 0.16 |
| Mottling at inclusion – no (%) | 115 [31] | 97 [26] | 0.57 |
| Mortality at day 90 | 154 [41] | 156 [42] | 0.88 |

## **Table S1 Baseline characteristics of study patients according to MAP target.**

*The target mean arterial pressure was 65 to 70 mmHg in the Low-MAP target group and 80 to 85 mmHg in the High-MAP target group.*

*COPD: Chronic obstructive pulmonary disease.*

*Values are represented as median [interquartile range].*

*The Simplified Acute Physiology Score (SAPS) II is based on 17 variables and scores range from 0 to 163, with a higher score indicating a more severe disease.*

*The score on the Sequential Organ Failure Assessment (SOFA) includes sub-scores ranging from 0 to 4 for each of five components (Circulation, lungs, liver, kidneys and coagulation). Aggregated scores range from 0 to 20, with higher scores indicating more severe organ failure.*

*Other sources of infection included blood, soft tissue, skin, central venous system, bones and joints, cardiac system, reproductive organs and unknown sources.*

*Acute kidney injury was defined as a renal SOFA score of 2 or more (plasma creatinine level > 1*.*9 mg per decilitre (168 µmol/L) or urinary output, < 500 mL per day).*

| **Characteristic** | **Low-MAP target**  n = 374 | **High-MAP target**  n = 373 | **p-value** |
| --- | --- | --- | --- |
| Median dose of norepinephrine – mg/24h [interquartile range] | | | |
| Day 1 | 16 (3-52) | 36 (14–91) | **<0**.**0001** |
| Day 2 | 2 (0-19) | 14 (1-48.25) | **<0**.**0001** |
| Day 3 | 0 (0–6) | 3.5 (0-23.25) | **<0**.**0001** |
| Day 4 | 0 (0-3) | 0.25 (0-15) | **<0**.**0001** |
| Day 5 | 0 (0-1) | 0 (0–11) | **0**.**0006** |
| Fluid intake – median [interquartile range] | | | |
| Day 1 | 3325 (2384-4520) | 3185 (2370-4207) | 0.33 |
| Day 2 | 2908 (2119-3809) | 2940 (2258-3735) | 0.71 |
| Day 3 | 2725 (2000-3543) | 2750 (2100-3565) | 0.47 |
| Day 4 | 2600 (2015-3574) | 2809 (2138-3628) | 0.22 |
| Day 5 | 2623 (1896-3476) | 2808 (2019-3319) | 0.41 |

## **Table S2 Evolution of norepinephrine doses and fluid intake according to the MAP target**

*The target mean arterial pressure was 65 to 70 mmHg in the Low-MAP target group and 80 to 85 mmHg in the High-MAP target group.*

| **Characteristics** | **No mottling**  (n = 451) | **Mottling**  (n = 296) | **p-value** |
| --- | --- | --- | --- |
| Death at day 28 - no (%) | 106 [23] | 144 [49] | <0**.**0001 |
| Death at day 90 - no (%) | 138 [31] | 172 [58] | <0**.**0001 |
| Dose of norepinephrine med– mg/24h (IQR 25-75) | | | |
| Day 1 | 17 [4-49] | 49 [19-106] | <0**.**0001 |
| Day 2 | 3 [0-22] | 18 [2-62] | <0**.**0001 |
| Day 3 | 0 [0-8.5] | 4 [0-31] | <0**.**0001 |
| Day 4 | 0 [0-5] | 1 [0-17] | <0**.**0001 |
| Day 5 | 0 [0-3] | 0 [0-12] | 0**.**01 |

## **Table S3 Outcomes according to the presence or absence of mottling at baseline or during the first 24 hours**

*Patients in the “no mottling” group had no mottling during the first 24 hours of septic shock. Patients in the mottling group had at least 2 hours of mottling during the first 24 hours of septic shock.*

|  | H24 | | | H48 | | | H72 | | |
| --- | --- | --- | --- | --- | --- | --- | --- | --- | --- |
|  | Low-MAP target | High- MAP target | p-value | Low-MAP target | High- MAP target | p-value | Low-MAP target | High- MAP target | p-value |
| Mottling monitoring available | n = 291 | n = 310 | - | n = 182 | n = 240 | - | n = 107 | n = 168 | - |
| With catecholamines | n = 250 | n = 290 | - | n = 143 | n = 207 |  | n = 81 | n = 142 | - |
| With mottling – n (%) | 61 (24) | 68 (23) | 0.84 | 35 (25) | 48 (20) | 0.79 | 18 (22) | 36 (25) | 0.63 |
| Without mottling – n (%) | 189 (76) | 220 (76) |  | 108 (59) | 159 (66) |  | 63 (78) | 106 (75) |  |
| Weaned from catecholamines | n = 41 | n = 20 | - | n = 39 | n = 33 | - | n = 26 | n = 26 | - |
| With mottling – n (%) | 0 | 0 | > 0.99 | 1 (3) | 0 | > 0.99 | 1 (4) | 0 (0) | > 0.99 |
| Without mottling – n (%) | 41 (100) | 20 (100) |  | 38 (97) | 33 (100) |  | 25 (100) | 26 (100) |  |
| Dead | n = 27 | n = 34 | - | n = 45 | n = 49 | - | n = 50 | n = 59 | - |
| No observation | n = 56 | n = 29 | - | n = 147 | n = 84 | - | n = 217 | n = 146 | - |

## **Table S4 Proportion of patients with mottling at H24, H48 and H72 of the inclusion.**

*Patients without observation (line “no observation”) were alive and weaned from catecholamine for more than 12 hours. As expected, the proportion of patients without observation increased more rapidly during follow-up in the Low- MAP target group because the duration of vasopressor treatment was shorter in this group.*
