# Supplementary material for: Effects of mean arterial pressure target on mottling and arterial lactate normalization in patients with septic shock: a post hoc analysis of the SEPSISPAM randomized trial
Source: Ann Intensive Care. 2022 Aug 19;12:78. doi: 10.1186/s13613-022-01053-1 (PMC9391564; doi:10.1186/s13613-022-01053-1)
Supplement: Supplementary file 2 — Additional file 2. Additional Figures. [file 13613_2022_1053_MOESM2_ESM.pptx]

## Slide 1
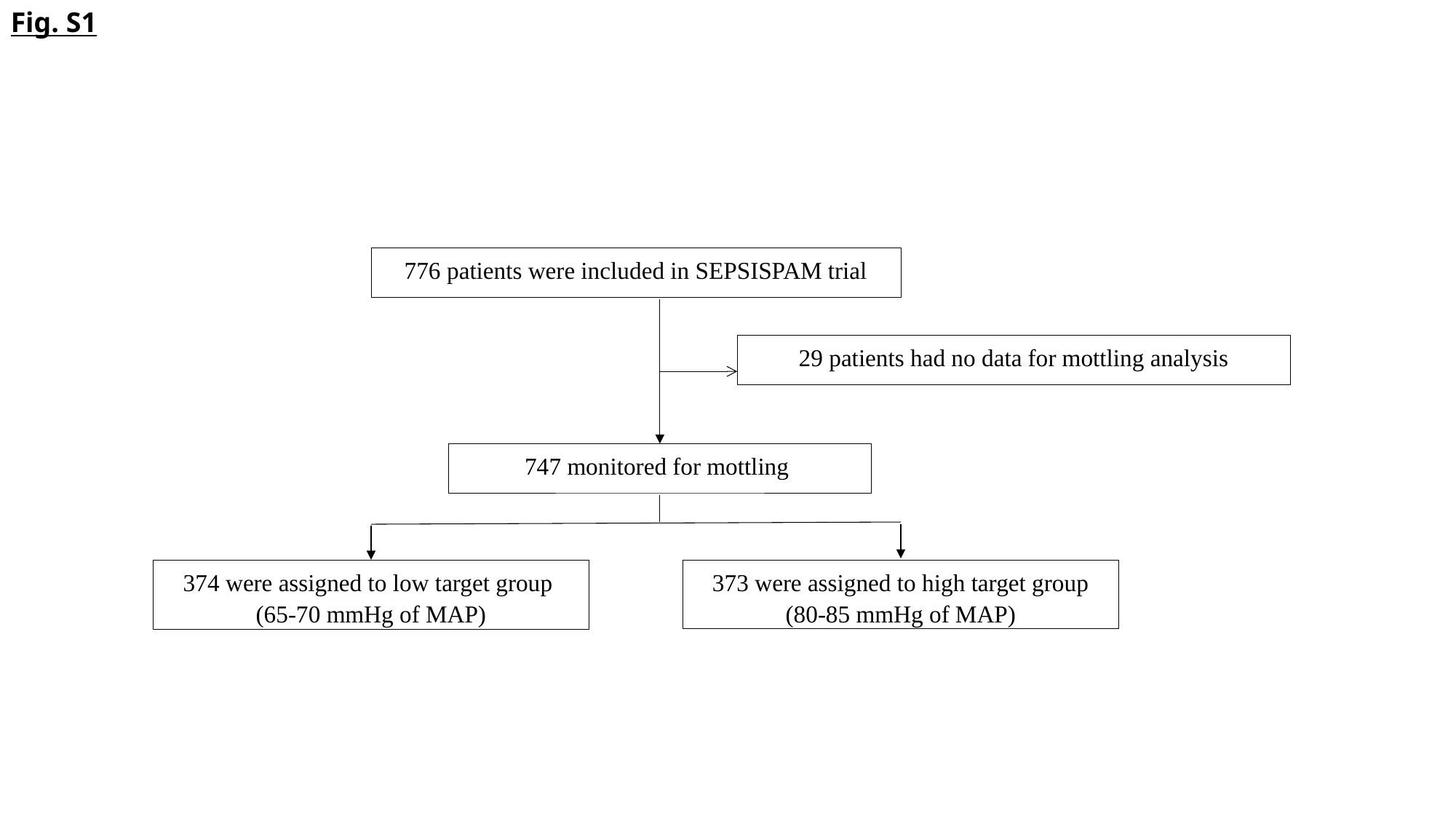

Fig. S1
776 patients were included in SEPSISPAM trial
29 patients had no data for mottling analysis
747 monitored for mottling
374 were assigned to low target group (65-70 mmHg of MAP)
373 were assigned to high target group (80-85 mmHg of MAP)

## Slide 2
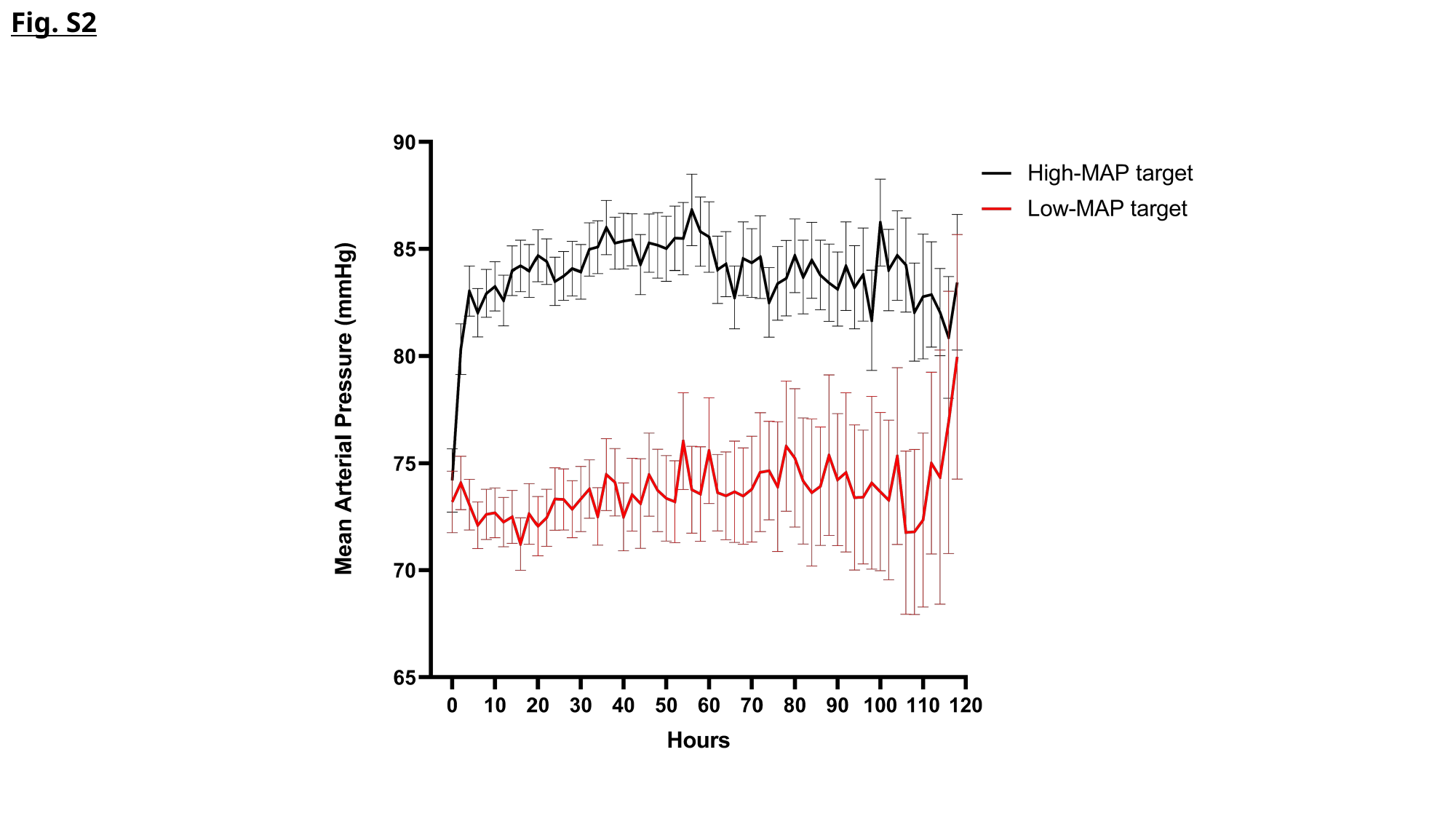

Fig. S2

## Slide 3
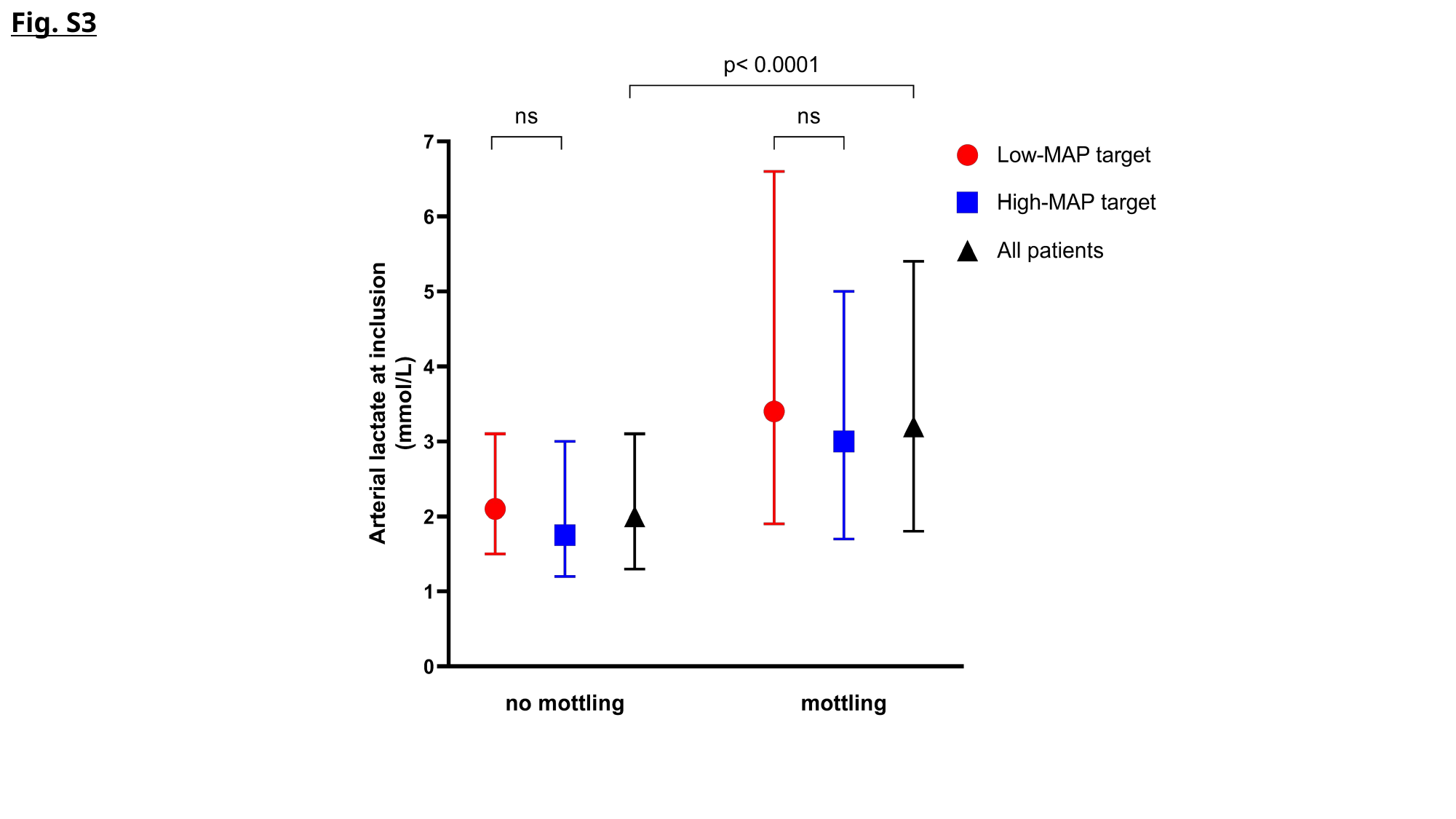

Fig. S3

## Slide 4
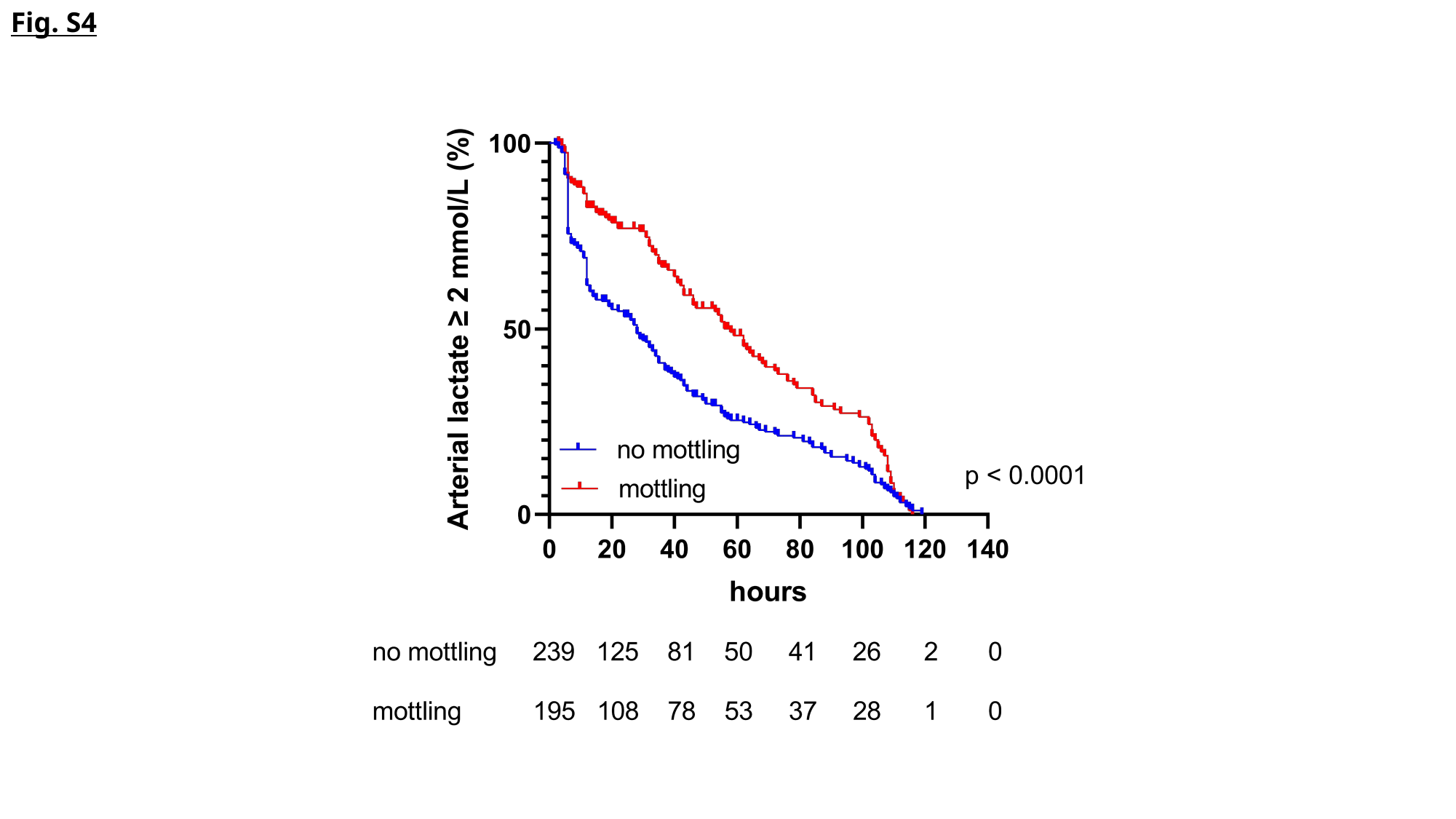

Fig. S4

## Slide 5
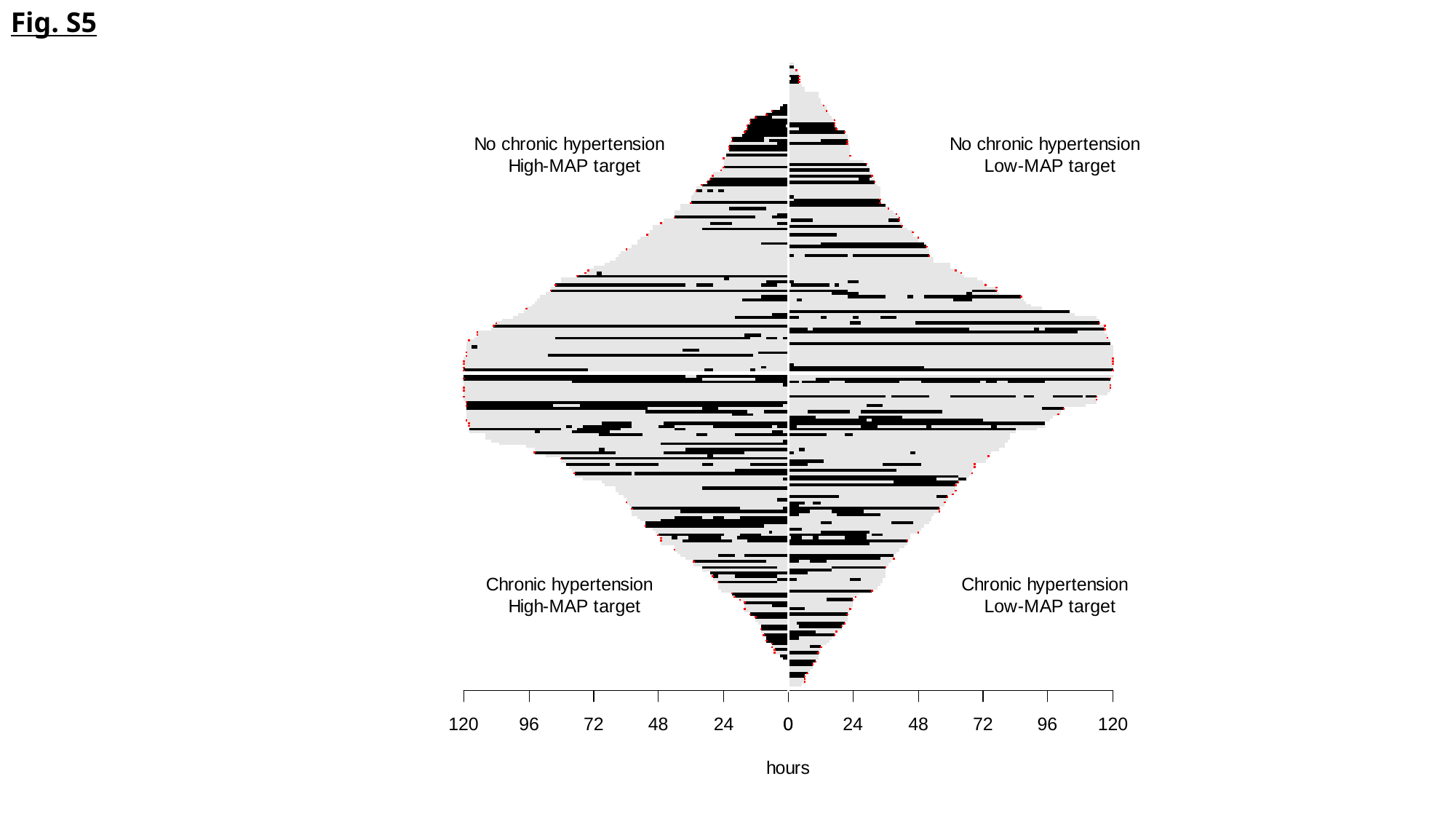

Fig. S5

## Slide 6
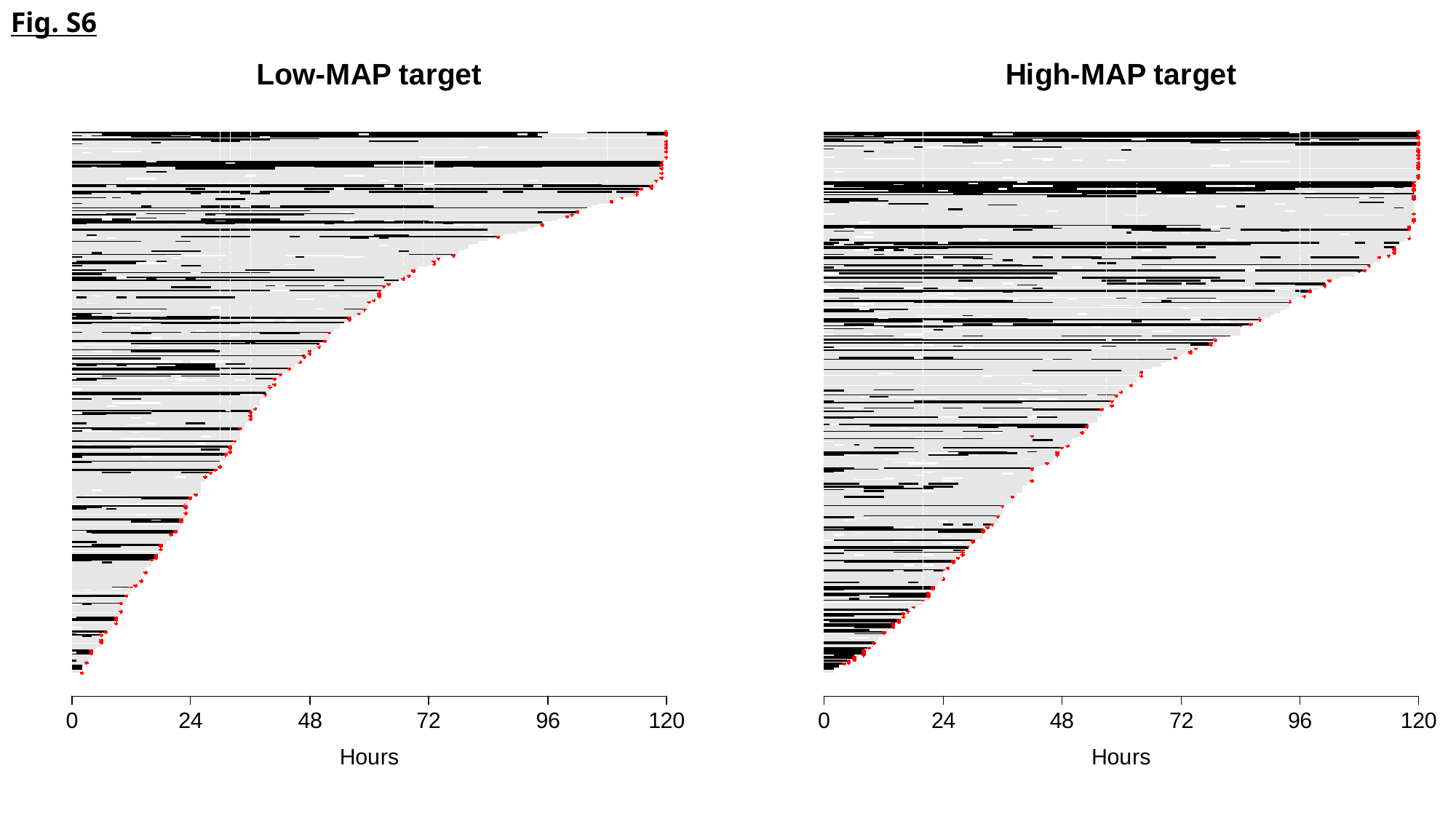

Fig. S6

## Slide 7
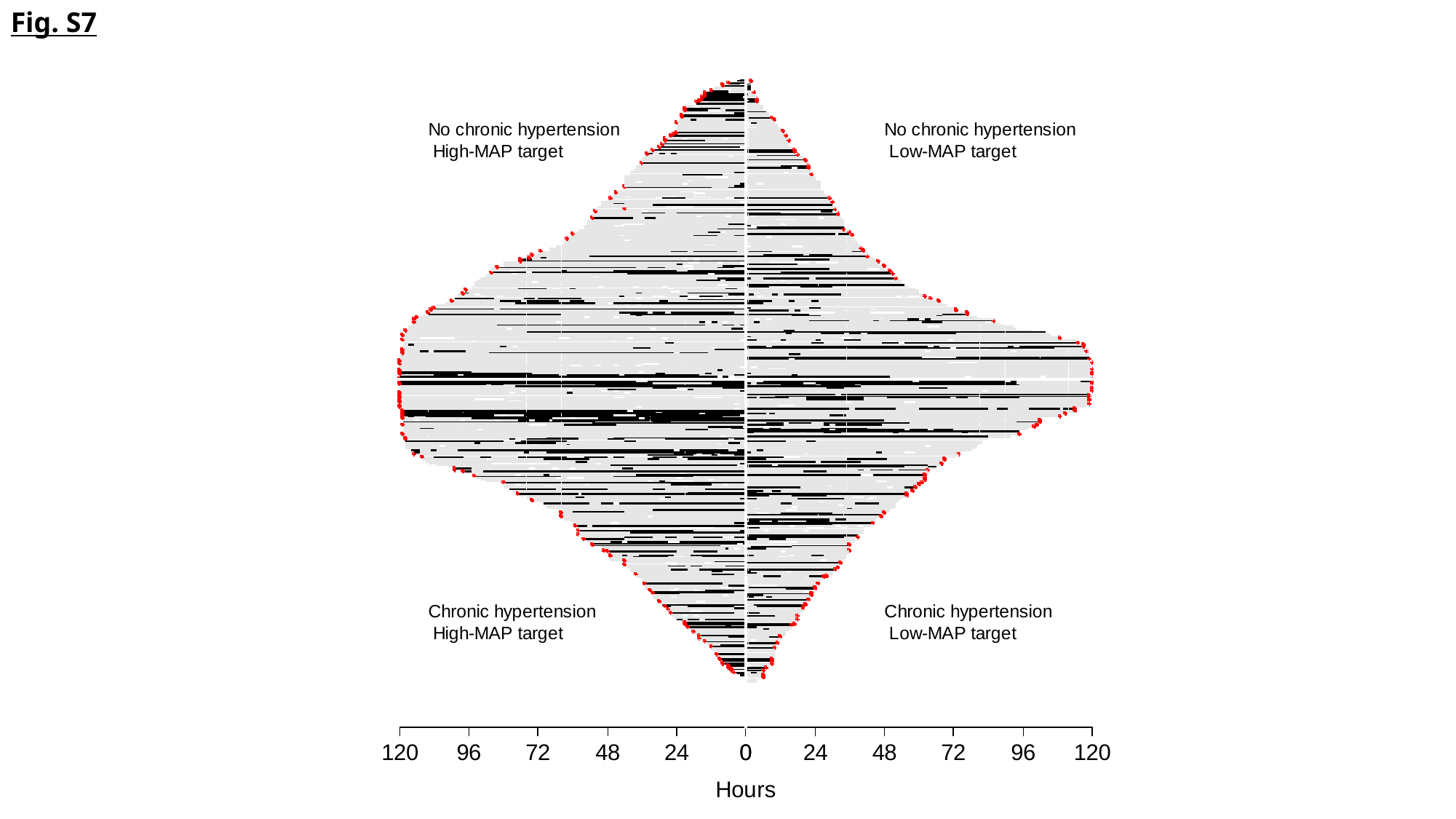

Fig. S7

## Slide 8
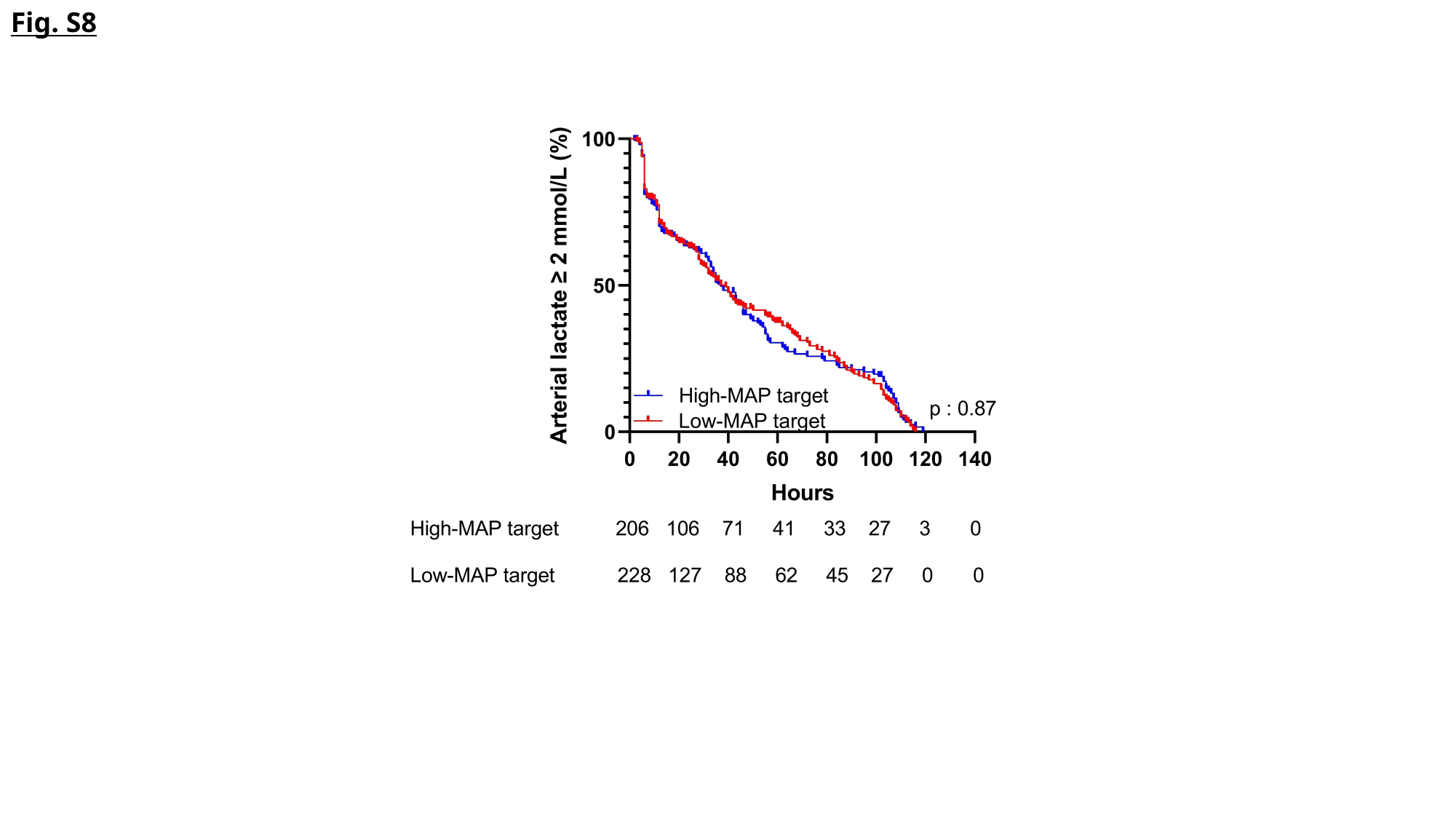

Fig. S8
